# A randomized trial of treatment for anterior cruciate ligament reconstruction by radial extracorporeal shock wave therapy

**DOI:** 10.1186/s12891-024-07177-8

**Published:** 2024-01-13

**Authors:** Yufeng Song, Xinle Che, Zheyun Wang, Mengshi Li, Runjie Zhang, Dongming Wang, Qiongfang Shi

**Affiliations:** 1 Second Hospital of Shanxi Medical University, Taiyuan, 030001 China; 2 Shanxi Medical University, Taiyuan, 030001 China; 3 Department of Rehabilitation, Xinghualing District Central Hospital, Taiyuan, 030001 China

**Keywords:** Anterior cruciate ligament reconstruction, Radial extracorporeal shock wave therapy, Rehabilitation

## Abstract

**Objective:**

The aim of this study was to explore the effects of radial extracorporeal shock wave therapy (rESWT) in patients with anterior cruciate ligament(ACL) reconstruction(ACLR).

**Methods:**

We conducted a randomized, controlled trial involving 72 eligible patients with ACL reconstruction in which we compared two strategies: the experimental group was standard rehabilitation plus rESWT and the control group was standard rehabilitation plus sham rESWT. The outcome was the change from baseline to 24 weeks in the average score on Lysholm knee joint score (LKS), range of motion (ROM), visual analogue scale (VAS) and International Knee Literature Committee (IKDC).

**Results:**

Of 36 subjects assigned to rehabilitation plus rESWT, 4 lost to follow up. Of 36 assigned to rehabilitation plus sham rESWT, 5 lost to follow up. The LKS, ROM and IKDC scores of the experimental group were markedly increased at 3 and 6 weeks after treatment (*P* < 0.001), and the VAS was notably decreased (*P* < 0.001). However, there were no significant differences in the LKS, ROM, IKDC and VAS between the groups at 24 weeks after treatment (*P* > 0.05).

**Conclusion:**

The strategy of rehabilitation plus rESWT had better functional outcomes after ACL reconstruction. As such, our study demonstrates that rESWT is essential for patients with ACL reconstruction. Early use of rESWT can improve joint function, pain relief and ability of daily living. rESWT has a positive effect on the overall rehabilitation of patients.

## Introduction

Anterior Cruciate Ligament Reconstruction (ACLR) is a usual treatment for patients with anterior cruciate ligament rupture. Effective ACL reconstruction requires professional rehabilitation to help patients return to their previously active lifestyles [1]. However, a few patients still have knee instability, decreased proprioceptive function, neuromuscular function, motor level difficult to restore and other problems. Therefore, clinical researchers must actively study new rehabilitation tools to help patients alleviate pain and restore neuromuscular function. Extracorporeal shock wave therapy (ESWT) is a kind of physical therapy, which generates mechanical waves and provides local therapeutic effects through the probe [2]. There are two types of ESWT generators, focused ESWT (fESWT) and radial ESWT (rESWT) [3]. Compared with fESWT, rESWT pressure increases slightly and much more slowly. When treated in the superficial area of interest, fESWT has more intensive energy exposure than rESWT. For this reason, the rESWT is considered less invasive than fESWT, so it might be more suitable for our study purposes [4].

Although rESWT is widely used for musculoskeletal disorders, the management of ACLR is unknown. Here, the purpose of this study was to explore whether rESWT contributed to the recovery of ACLR.

## Materials and methods

### Study design and participants

**Fig. 1:**
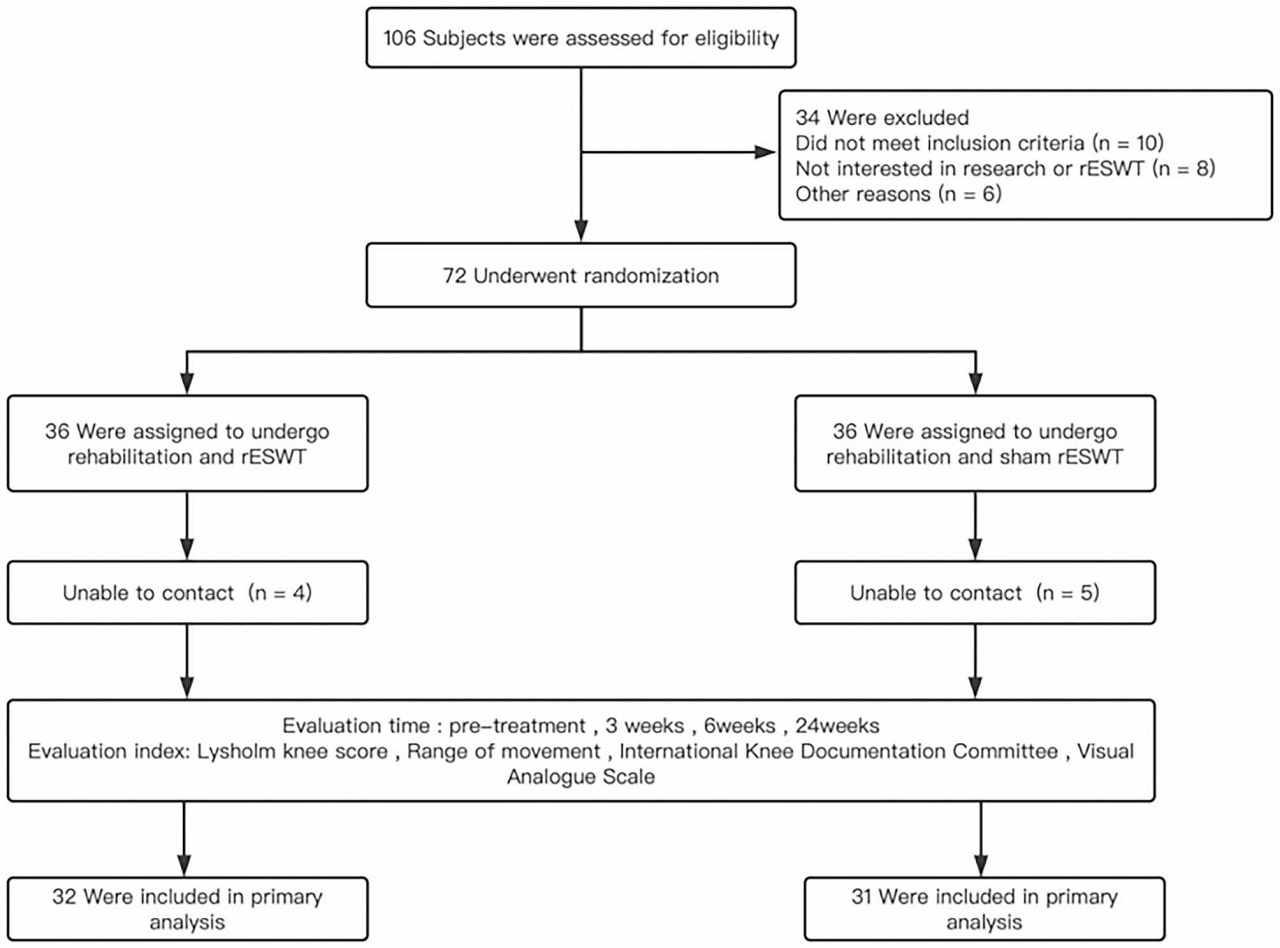
Flowchart of the study design

We conducted the randomized, single-blind clinical trial in China, with subjects recruited from November 2021 to March 2023 in the Second Hospital of Shanxi Medical University. Patients were divided into two groups using the software program. The experimental group : standard rehabilitation plus rESWT; The control group: standard rehabilitation plus sham rESWT (Fig. 1). Sham shock wave therapy refers to the use of a simulated therapeutic head only to reduce interference brought on by vibration during shock wave therapy. It did not produce sound waves and had no therapeutic effect.

### Inclusion and exclusion criteria

The inclusion criteria: (i) age ≥ 18years; (ii) unilateral ACL rupture without other ligament or meniscus injury; (iii) no other osteoarticular or soft tissue lesions in the lower extremities; (iv) no cognitive impairment or disturbance of consciousness. In addition,subjects should have no contraindications to shock waves to ensure the trial goes smoothly.

The exclusion criteria: (i) patients with tumors or other serious diseases; (ii) history of deep vein thrombosis or vascular pathology in any lower limb; (iii) rheumatoid arthritis or other signifcant co-morbidities; (iv) intraarticular injections into the knee in the preceding 6 months.

### Interventions

#### Preoperative rehabilitation

To improve the outcome of postoperative conditions, patients undergoing ACL reconstruction should undergo rehabilitation before surgery [5, 6]. Patients who are better prepared, both psychologically and functionally, prior to ACLR also have better outcomes after ACLR. Preoperative care was personalized according to the subject’s situation, covering preoperative assessment, rehabilitation guidance and psychological counseling. Deficits in passive joint range of motion and quadriceps strength should be specifically targeted because these factors are associated with Postoperative results [7]. A common belief is that preoperative rehabilitation has been associated with better postoperative function and activity level compared. To reduce the risk of knee re-injury, clinicians should provide education to patients, including the possible benefits of rehabilitation.

#### Standard rehabilitation programs

Phase 1: For the treatment of completely passive stretching, quadriceps femoris functional exercise should be started on the first day after ACL reconstruction. Cryotherapy can be used to treat the pain during training. Active and passive range-of-motion exercises (e.g., straight leg raise in the supine position and lateral leg raise, heel slides and continuous passive motion (CPM) was used to carry out joint movement), and it is advocated to manage effusion by adjusting the load in this phase. After surgery, both weight-bearing (closed kinetic chain) and non-weight-bearing (open kinetic chain) exercises were performed simultaneously.

Phase 2: This phase will start neuromuscular training and muscle strength training. The purpose of neuromuscular training is to improve the dynamic stability of the knee joint by establishing more beneficial proprioception and motor control strategies [7]. The goal of muscle strength training is to restore the muscle strength needed by patients to participate in sports and activities of daily living. Muscle strength exercises will gradually develop from a lighter load with a higher number of repetitions to a heavier load with fewer repetitions. If the patient can perform two additional repetitions according to the target repetition times, the load will be increased in the next training [8]. The gradual increase in training load is also a key component of the transition to activity/movement [9].

Phase 3: Individualized training should be carried out in the late rehabilitation according to the specific goals and sports needs of patients. Generally, this stage includes specific damage of heavy strength training, strength and agility training, and specific exercise training. When patients gradually return to exercise, all kinds of pivoting sports are effective injury prevention plans, including lower limb strength exercises and low-risk exercise mode training. Knee joint effusion and knee joint pain rules are commonly used clinical indicators to evaluate response to load [10].

#### rESWT programs

**Fig. 2:**
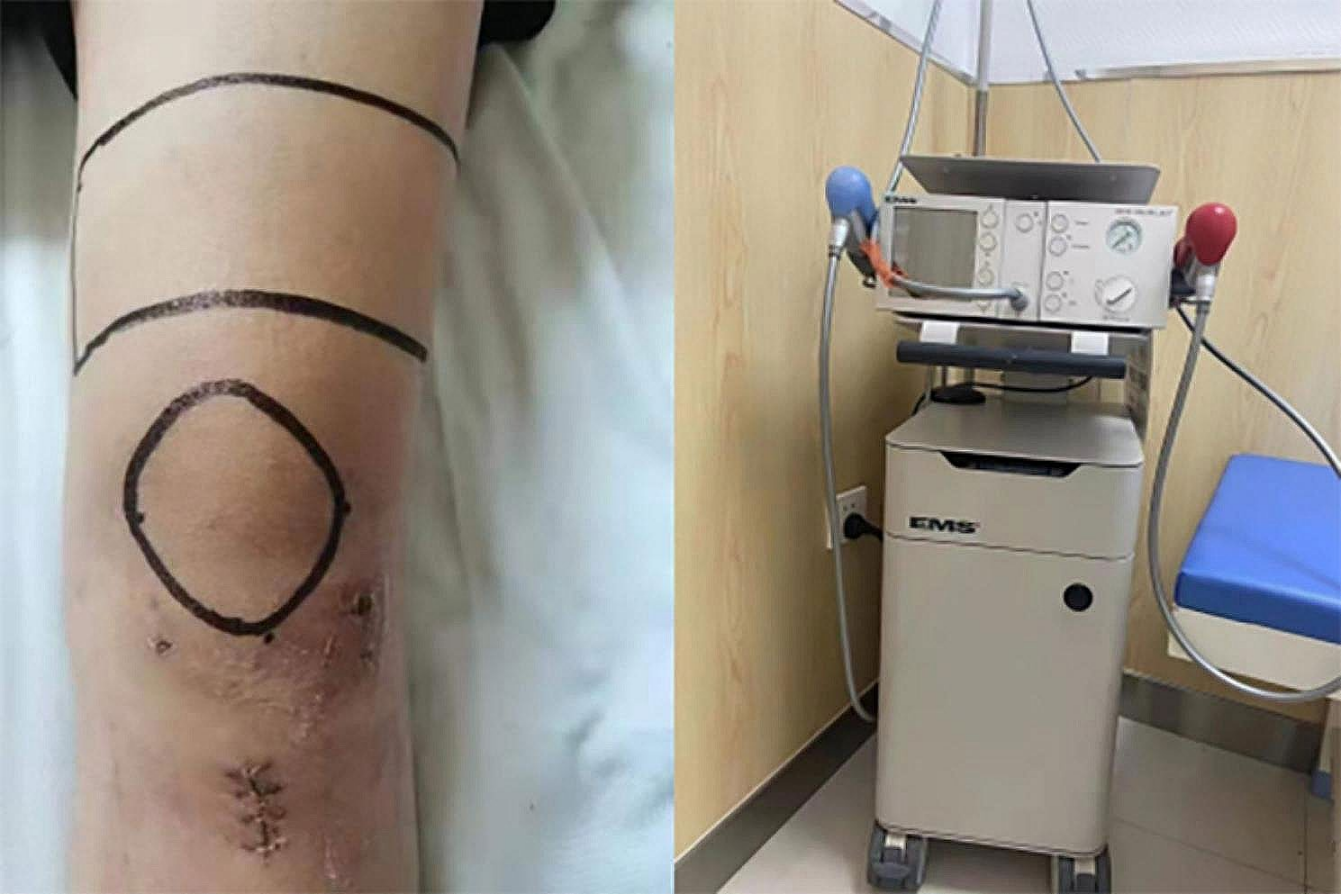
Treatment area of radial extracorporeal shock wave therapy (rESWT)

The experimental group received rESWT and standard rehabilitation, while the control group received sham rESWT and standard rehabilitation. Patients in the experimental group started receiving rESWT on the second postoperative day, and the standard rehabilitation training program was the same in both groups. The source of shockwave was from Swiss Dolor Clast (EMS, Switzerland). The shockwave was focused at the area around the patella and the rectangular area 10 cm above the upper edge of the patella, avoiding the operation area (Fig. 2). Application of 2500 impulses of ESWT at a frequency of 6–8 Hz (0.298 mJ/mm2) was administered to the treatment area. The treatment schedule was 6 consecutive weeks, once a week. If the patient cannot tolerate rESWT due to pain during treatment, the intensity is appropriately reduced to a low energy flux density range (0.08–0.28 mJ/mm2) and the shock is reduced to 1200–1500. Sham rESWT was performed with a simulated head (consistent with the appearance of real head but inneffective) to the control group.

### Outcome measures

The baseline data were recorded before treatment and followed up at the 3rd, 6th and 24th weeks. The Visual Analogue Scale (VAS), Lysholm Knee Score (LKS), Range of movement (ROM)and the International Knee Documentation Committee (IKDC) were adopted to evaluate the changes in pain and function of patients in both groups. These four scales are internationally recognized to evaluate knee function and frequently used in clinical studies.

### Statistical analysis and plotting

The patients’ baseline demographics and knee-related characteristics among the designated groups were expressed as mean ± standard deviation (SD), and baseline characteristics were tested by independent t-test and chi-square test, the Mann-Whitney U test was used for group comparisons of functional outcomes. All data analyses were carried out using the SPSS statistical software version 26.0, and the statistical significance of all tests was evaluated at a predetermined significance level of 0.05. In addition, plots were made with Originpro8.1.

## Results

### Baseline characteristics

Nine participants were lost before completing the study protocol due to being unable to contact them, leaving 63 completed participants. There were no statistically significant differences between groups in age, sex,side of injury, or cause of injury (Table 1). Therefore, two groups are comparable. Meanwhile, there were no adverse events reported.

**Table 1:** Comparison of baseline characteristics between the two groups

Demographics	experimental group (*n* = 32)	control group (*n* = 31)	P-values
Age(y)	27.94 ± 6.38	27.00 ± 6.11	0.628
Male sex, n(%)	21(65.63)	18(58.06)	0.537
Side of injury (left/right)	15/17	16/15	0.707
exercise	15	14	0.870
Car accidents	9	10	
Fall	5	3	
Other	3	4	

### Lysholm knee score

**Fig. 3:**
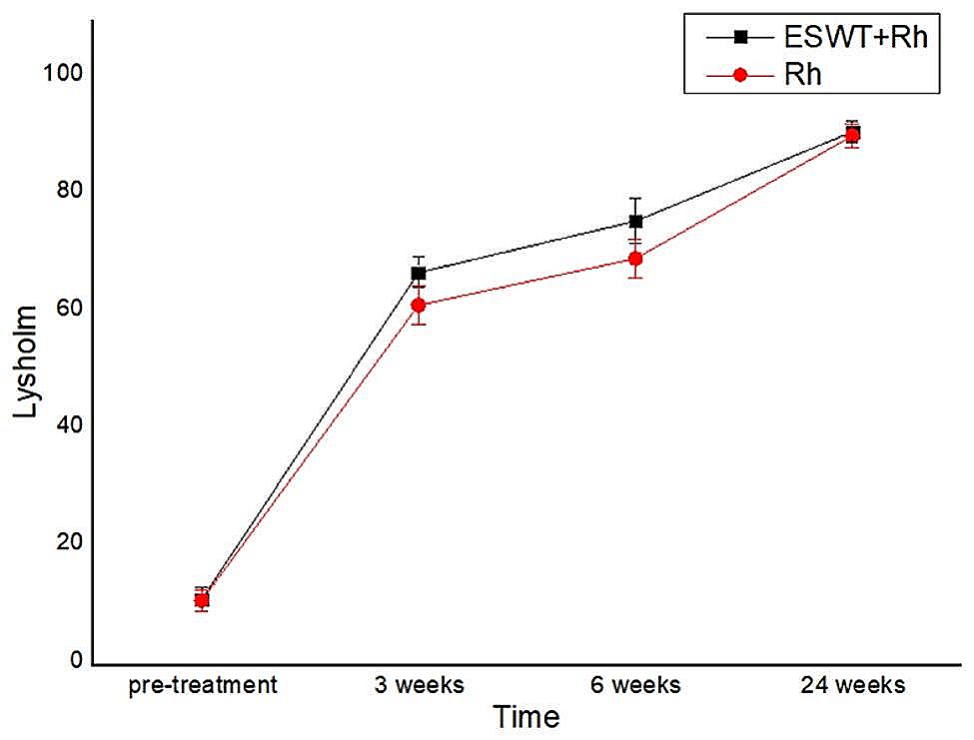
Change in LKS over the duration of the study

At pre-treatment, there were no statistically significant differences between the experimental and control groups in terms of LKS. Interestingly, the LKS was significantly different in the two groups at 3 and 6 weeks after treatment. The LKS of the two groups continued to increase from pre-treatment to 24 weeks after treatment, but there was no significant difference between the two groups at 24 weeks after treatment (*P* > 0.05)(Table 2; Fig. 3).

**Table 2:** Change in LKS over the duration of the study

Group	Cases	pre-treatment	3 week after treatment	6 week after treatment	24 week after treatment	P-values
experimental group	32	11.19 ± 2.023	66.91 ± 2.607	75.69 ± 3.814	90.87 ± 1.792	0.038
control group	31	11.00 ± 1.751	61.35 ± 3.179	69.29 ± 3.268	90.19 ± 2.040	
*P*-values		0.646	< 0.001	< 0.001	0.224	

### Range of movement

**Fig. 4:**
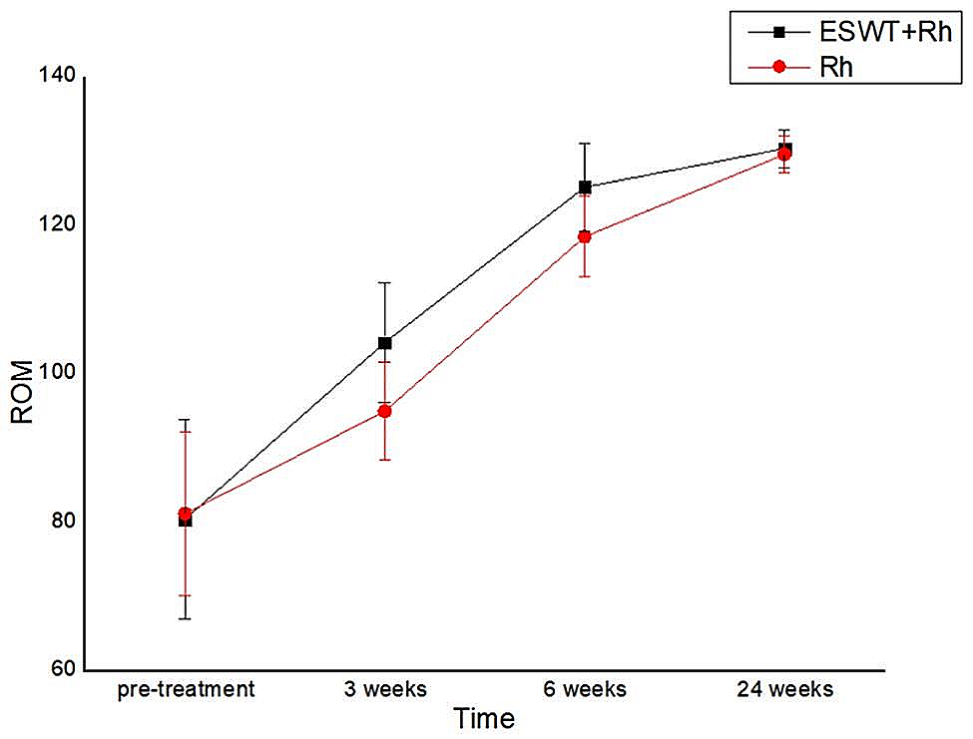
Change in ROM over the duration of the study

There were no statistically significant differences between groups in terms of ROM at pre-treatment. Interestingly, the ROM was significantly different in the experimental and control groups at 3 and 6 weeks after treatment. Conservely, there was no significant difference in ROM between groups at 24 weeks after treatment (*P* > 0.05)(Table 3; Fig. 4).

**Table 3:** Change in ROM over the duration of the study

Group	Cases	pre-treatment	3 week after treatment	6 week after treatment	24 week after treatment	P-values
experimental group	32	80.41 ± 13.423	104.22 ± 8.091	125.16 ± 5.941	130.34 ± 2.509	0.050
control group	31	81.19 ± 11.071	94.97 ± 6.631	118.48 ± 5.464	129.55 ± 2.514	
P-values		0.794	< 0.001	< 0.001	0.348	

### International knee documentation committee

**Fig. 5:**
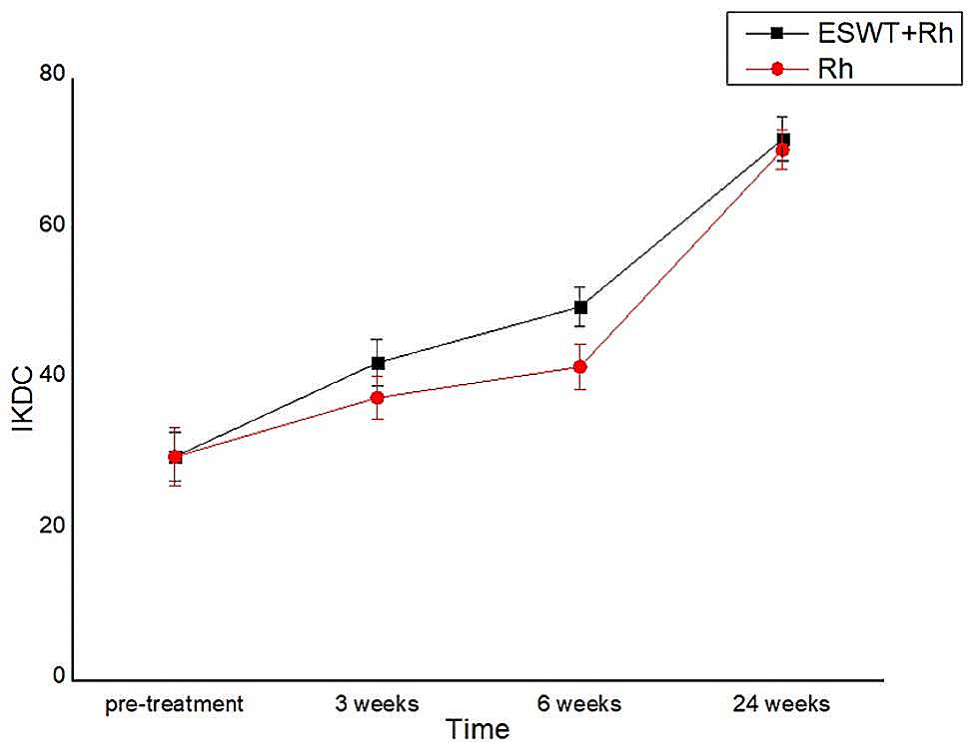
Change in IKDC over the duration of the study. Data are presented as mean ± SD

At pre-treatment, there were no significant differences between the experimental and control groups in terms of IKDC. At 3 and 6 weeks after treatment, both groups experienced significant increases in IKDC with group differences. Of note, there was no significant difference in IKDC between groups at 24 weeks after treatment (*P* > 0.05)(Table 4; Fig. 5).

**Table 4:** Change in IKDC over the duration of the study

Group	Cases	pre-treatment	3 week after treatment	6 week after treatment	24 week after treatment	P-values
experimental group	32	29.78 ± 3.170	42.25 ± 3.111	49.69 ± 2.596	71.94 ± 2.940	0.008
control group	31	29.77 ± 3.939	37.61 ± 2.895	41.71 ± 3.079	70.55 ± 2.593	
P-values		0.879	< 0.001	< 0.001	0.055	

### Visual analogue scale

**Fig. 6:**
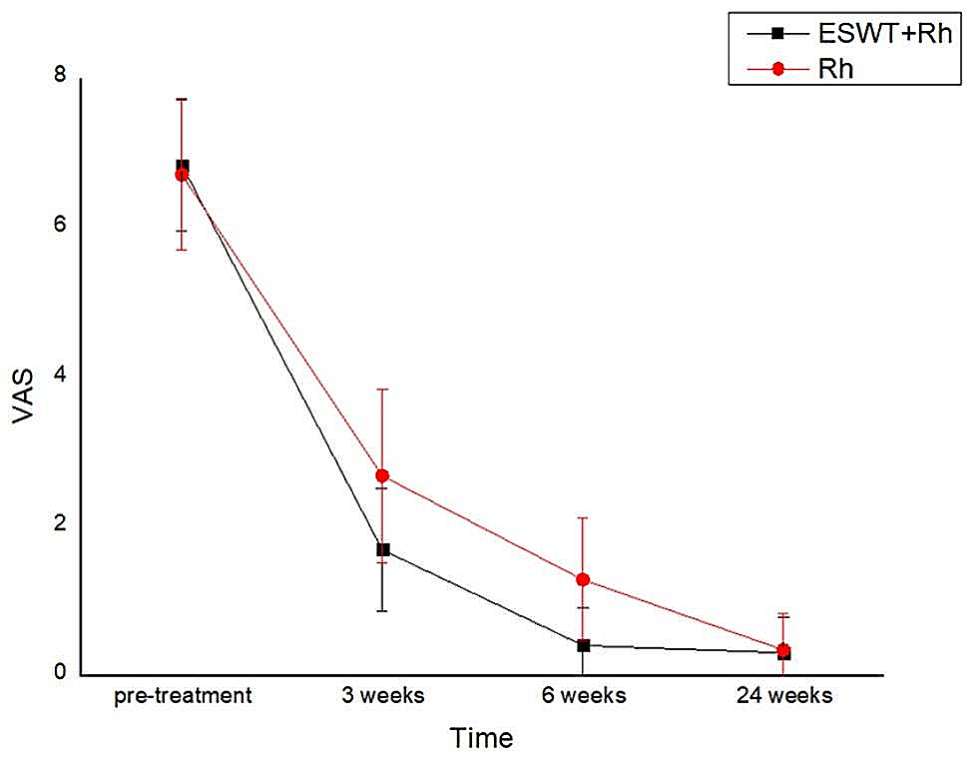
Change in VAS over the duration of the study

There were no significant differences in VAS at pre-treatment. Importantly, we found that, compared with the control group, the VAS in the experimental group was significantly lower at 3 weeks and 6 weeks after treatment. At the same time, no significant changes were found between the two groups at 24 weeks after treatment (*P* > 0.05)(Table 5; Fig. 6).

**Table 5:** Change in VAS over the duration of the study

Group	Cases	pre-treatment	3 week after treatment	6 week after treatment	24 week after treatment	P-values
experimental group	32	6.84 ± 0.884	1.69 ± 0.821	0.41 ± 0.499	0.31 ± 0.471	0.033
control group	31	6.71 ± 1.006	2.68 ± 1.166	1.29 ± 0.824	0.35 ± 0.486	
P-values		0.560	0.001	< 0.001	0.724	

## Discussion

This study was to examine the effect of rESWT on muscle strength, physical function, knee pain and range of motion during an ACLR rehabilitation program. The results of this randomized, controlled trial involving ACL reconstruction indicate that a strategy of rESWT plus standard rehabilitation was superior to a strategy of standard rehabilitation.

After ACL reconstruction, there may be changes in knee joint control and motor perception, decreased spinal reflex and excitability of corticospinal pathway, and persistent defects in quadriceps function [11, 12]. Therefore, early intervention in rehabilitation therapy is very important to improve the postoperative effect. The traditional initial treatment of choice is standard rehabilitation, comprehending modalities such as cryotherapy, continuous passive motion(CPM) [13], high-intensity neuromuscular electrical stimulation (NMES) [14], neuromuscular training and muscle strength training [7]. However, this method has not produced encouraging results over the past years.

To our knowledge, there is limited evidence on functional outcomes of the ACLR by using rESWT. This is a study to compare the rESWT program and standard rehabilitation program for patients with ACLR. rESWT acts at the tendon-bone interface by physical impact, triggering cell regeneration and stimulating the release of growth factors [15–17]. The effect of physical energy on biological tissues is similar to a cascade process, in which the energy of shock wave sequentially activates the cytoskeletal system and organelles, releasing proteins for the healing process [18]. Among these, the growth factor stimulates cell surface to express the protein, activating the intercellular interactions. In addition, rESWT promoted extracellular matrix metabolism, neovascularization [19], bone mineralization, and formation. Reductions in adhesions and improvements in joint mobility allow patients to recover better, including reductions in pain and improvements in function and microcirculation.

In the present study, we sought to preliminarily determine the efficacy of rESWT in reducing pain and improving function and mobility in ACL reconstruction patients. Additionally, VAS, IKDC, LKS and ROM were assessed. In the course of rehabilitation, pain, inflammatory irritation and limited mobility [20]all have an adverse impact on patients’ mood and training, as well as the overall rehabilitation outcome. Pain mainly originates from inflammation, swelling and muscle adhesions in rehabilitation [21]. The VAS score is mainly used to score the patients’ pain and quantify subjective perception. In our study, there was a more pronounced improvement in VAS of experimental group at 3 and 6 weeks (*P* < 0.05). The reasons for the outcome were that standard rehabilitation stimulates knee mechanoreceptors by joint mobilization and traction, which achieve the effect of assisted movement, only in a movable range. In contrast, rESWT can modulate local inflammatory factors and nociceptive transmission, exert anti-inflammatory, anti-swelling, and analgesic effects, and make the pain-relieving effects more pronounced [22].

The IKDC primarily assesses activities of daily living following knee injury, and provides a comprehensive assessment of stability and pain [23]. The higher the score, the lower the subjective discomfort and the stronger the function. The improvement in IKDC of experimental group (*P* < 0.05) at 3 and 6 weeks might be attributed to a decrease of postoperative discomfort. Even though rESWT point had the highest mean IKDC score improvement after 24 weeks the differences between the groups were statistically insignificant. However, due to the considerable disparity in the patients’ tolerance, the questionnaire ratings may be influenced.

Lysholm score emphasizes patients’ subjective feelings about symptoms, and combined with digital score and patients’ daily activity level, the degree of patients’ dysfunction can be graded. The research shows that the scale is the most reliable for patients with anterior cruciate ligament reconstruction, and the score difference is more significant when evaluating patients with self-limiting activities. Wang et al [24]reported an rESWT treatment to ACL reconstruction patients in the bone tunnel area, and the rESWT group showed higher LKS and superior knee stability at a follow-up of 2 years after surgery, similar to our results of LKS in the experimental group, all dramatically higher than the control group (*P* < 0.05). In addition, ROM can determine the degree of joint limitation as an evaluation method for treatment and training. Notably, the results of ROM were significantly higher in the experimental group at 3 and 6 weeks (*P* < 0.05), indicating a pronounced improvement in knee function. After ACL reconstruction, the injured site secretes growth factors and cytokines, which guide cells to migrate from the periphery of the graft to the injured site, further proliferate and produce extracellular matrix, and tendinous differentiation is performed under growth factor stimulation to promote ligament healing. In this study, rESWT combined with standard rehabilitation methods had better recovery effect, which was consistent with Lu et al. [25] who found that rESWT could enhance the residual cell activity of anterior cruciate ligament and the activity and differentiation of surrounding cells, induce ACL cells to secrete transforming growth factor TGF-βand vascular endothelial growth factor VEGF, and promote vascular and tissue regeneration. Experimental group had a larger range of ROM, and the LKS was higher than that of the control group. The reason may be that standard rehabilitation methods can release adhesion, improve physiological axial movement, whereas rESWT enhances ACL residual cell activity, strengthen tendon-bone connection, significantly improve ligament recovery, stimulate muscles around the knee joint, improve local lymphatic circulation, and promote inflammatory absorption [26].

In the present research, we have demonstrated that all participants showed significant improvement in knee function after 24 weeks of treatment. Experimental design in this study focused on the effects of rESWT on pain relief, knee function and mobility in the short-term postoperative period, with follow-up and data recorded at 3, 6 and 24 weeks. Some measures about subjective perception and knee joint function have been improved in different degrees, which is consistent with the research results of Aldajah et al. [27]. This study found that rESWT can significantly relieve the pain, upper limb function and grip strength in volunteers with humeral epicondylitis. According to Lie et al [28], the application of rESWT triggered nerve tissue regeneration, stimulated cell differentiation, and reduced neuronal loss, all of these might aid to repair acute traumatic spinal cord damage. Because early postoperative rehabilitation training is particularly important for the recovery of knee joint function after ACL reconstruction, it is more vital to study the improvement of knee joint function and rehabilitation effect by rESWT in the short term after surgery for early rehabilitation training.

### Strengths and limitations

rESWT, as a positive factor affecting the rapid recovery of patients, has the advantages of non-invasive, low cost and energy regulation, and can strengthen the training and auxiliary effect in the postoperative rehabilitation stage. The results of this study suggest that rESWT is promising in the rehabilitation of ACL reconstruction and musculoskeletal system diseases, therefore, it is worthy of further study in this direction. However, this study is not without its limitations. Our study did not provide a more objective evaluation of healing, such as MRI, X-ray, KT1000, etc. Furthermore, it remains unclear whether patients with higher pain levels and severe knee impairment would benefit from rESWT and whether multiple applications of rESWT would lead to different outcomes. Due to the limitations of this study, it is necessary to include more comprehensive evaluation criteria in subsequent studies. For patients after ACL reconstruction, the optimal treatment protocol has not yet been established, and a higher level of evidence is needed to demonstrate and determine the efficacy of rESWT in future clinical trials.

## Conclusion

The randomized controlled trial demonstrated that the individuals showed varying degrees of improvement in pain relief and knee function after 24 weeks of rehabilitation training. rESWT can regulate local inflammatory factors and nociceptive transmission, acting as an anti-inflammatory, anti-swelling and analgesic, it can also promotes ACL residual cell activity, strengthens tendon-bone connections, improves ligament repair, stimulates peri-knee muscles and local lymphatic circulation around the knee, to achieve the recovery of improving knee function and mobility. Taken together, the application of rESWT in the early rehabilitation period after ACL reconstruction is an effective and positive method, and this method can reduce the pain level of subjects and improve their knee joint function.

## Data Availability

The datasets used and/or analysed during the current study are available from the corresponding author on reasonable request.

## Abbreviations

rESWT: Radial Extracorporeal shock wave therapy
ACL: Anterior Cruciate Ligament
LKS: Lysholm knee score
ROM: Range of movement
VAS: Visual Analogue Scale
IKDC: International Knee Documentation Committee

## References

[1] Brinlee AW, Dickenson SB, Hunter-Giordano A (2022). ACL Reconstruction Rehabilitation: Clinical Data, Biologic Healing, and Criterion-based milestones to inform a return-to-Sport Guideline[J]. Sports Health.

[2] Chen T, Zhang P, Chen J (2017). Long-term outcomes of Anterior Cruciate Ligament Reconstruction using either synthetics with Remnant Preservation or Hamstring autografts: a 10-Year longitudinal Study[J]. Am J Sports Med.

[3] Notarnicola A, Moretti B (2012). The biological effects of extracorporeal shock wave therapy (eswt) on tendon tissue[J]. Muscles Ligaments Tendons J.

[4] Dymarek R, Halski T, Ptaszkowski K (2014). Extracorporeal shock wave therapy as an adjunct wound treatment: a systematic review of the literature[J]. Ostomy Wound Manage.

[5] Grindem H, Granan LP, Risberg MA (2015). How does a combined preoperative and postoperative rehabilitation programme influence the outcome of ACL reconstruction 2 years after surgery? A comparison between patients in the Delaware-Oslo ACL Cohort and the Norwegian national knee Ligament Registry[J]. Br J Sports Med.

[6] Shaarani SR, O’Hare C, Quinn A (2013). Effect of prehabilitation on the outcome of anterior cruciate ligament reconstruction[J]. Am J Sports Med.

[7] van Melick N, van Cingel RE, Brooijmans F (2016). Evidence-based clinical practice update: practice guidelines for anterior cruciate ligament rehabilitation based on a systematic review and multidisciplinary consensus[J]. Br J Sports Med.

[8] Eitzen I, Moksnes H, Snyder-Mackler L (2010). A progressive 5-week exercise therapy program leads to significant improvement in knee function early after anterior cruciate ligament injury[J]. J Orthop Sports Phys Ther.

[9] Blanch P, Gabbett TJ (2016). Has the athlete trained enough to return to play safely? The acute:chronic workload ratio permits clinicians to quantify a player’s risk of subsequent injury[J]. Br J Sports Med.

[10] Adams D, Logerstedt DS, Hunter-Giordano A (2012). Current concepts for anterior cruciate ligament reconstruction: a criterion-based rehabilitation progression[J]. J Orthop Sports Phys Ther.

[11] Gatewood CT, Tran AA, Dragoo JL (2017). The efficacy of post-operative devices following knee arthroscopic surgery: a systematic review[J]. Knee Surg Sports Traumatol Arthrosc.

[12] An YW, DiTrani LA, Lehmann T (2019). Neuroplastic changes in anterior cruciate ligament reconstruction patients from neuromechanical decoupling[J]. Scand J Med Sci Sports.

[13] Auersperg V, Trieb K (2020). Extracorporeal shock wave therapy: an update[J]. EFORT Open Rev.

[14] Kim KM, Croy T, Hertel J (2010). Effects of neuromuscular electrical stimulation after anterior cruciate ligament reconstruction on quadriceps strength, function, and patient-oriented outcomes: a systematic review[J]. J Orthop Sports Phys Ther.

[15] Park C, Lee S, Yi CW (2015). The effects of extracorporeal shock wave therapy on frozen shoulder patients’ pain and functions[J]. J Phys Ther Sci.

[16] Lee S, Lee S, Jeong M (2017). The effects of extracorporeal shock wave therapy on pain and range of motion in patients with adhesive capsulitis[J]. J Phys Ther Sci.

[17] Xu Y, Wu K, Liu Y (2019). The effect of extracorporeal shock wave therapy on the treatment of moderate to severe knee osteoarthritis and cartilage lesion[J]. Med (Baltim).

[18] Mittermayr R, Haffner N, Feichtinger X et al. The role of shockwaves in the enhancement of bone repair - from basic principles to clinical application[J]. Injury, 2021;52(Suppl 2):S84–S90. 10.1016/j.injury.2021.02.081 33714550

[19] Fu M, Sun CK, Lin YC (2011). Extracorporeal shock wave therapy reverses ischemia-related left ventricular dysfunction and remodeling: molecular-cellular and functional assessment[J]. PLoS ONE.

[20] Lepley AS, Gribble PA, Thomas AC (2015). Quadriceps neural alterations in anterior cruciate ligament reconstructed patients: a 6-month longitudinal investigation[J]. Scand J Med Sci Sports.

[21] Sepúlveda F, Sánchez L, Amy E (2017). Anterior cruciate ligament Injury: return to play, function and long-term Considerations[J]. Curr Sports Med Rep.

[22] Storheim K, Gjersing L, Bølstad K (2010). [Extracorporeal shock wave therapy (ESWT) and radial extracorporeal shock wave therapy (rESWT) in chronic musculoskeletal pain][J]. Tidsskr nor Laegeforen.

[23] Hefti F, Müller W, Jakob RP (1993). Evaluation of knee ligament injuries with the IKDC form[J]. Knee Surg Sports Traumatol Arthrosc.

[24] Wang CJ, Ko JY, Chou WY (2014). Shockwave therapy improves anterior cruciate ligament reconstruction[J]. J Surg Res.

[25] Lu CC, Chou SH, Shen PC (2020). Extracorporeal shock wave promotes activation of anterior cruciate ligament remnant cells and their paracrine regulation of bone marrow stromal cells’ proliferation, migration, collagen synthesis, and differentiation[J]. Bone Joint Res.

[26] Murray MM, Magarian E, Zurakowski D (2010). Bone-to-bone fixation enhances functional healing of the porcine anterior cruciate ligament using a collagen-platelet composite[J]. Arthroscopy.

[27] Aldajah S, Alashram AR, Annino G et al. Analgesic effect of extracorporeal shock-Wave Therapy in individuals with lateral epicondylitis: a randomized controlled Trial[J]. J Funct Morphol Kinesiol, 2022;7(1). 10.3390/jfmk7010029 PMC8955256 35323612

[28] Leister I, Mittermayr R, Mattiassich G (2022). The effect of extracorporeal shock wave therapy in acute traumatic spinal cord injury on motor and sensory function within 6 months post-injury: a study protocol for a two-arm three-stage adaptive, prospective, multi-center, randomized, blinded, placebo-controlled clinical trial[J]. Trials.

